# Acknowledgment to Reviewers of *EJIHPE* in 2021

**DOI:** 10.3390/ejihpe12020013

**Published:** 2022-01-30

**Authors:** EJIHPE Editorial Office

**Affiliations:** MDPI AG, St. Alban-Anlage 66, 4052 Basel, Switzerland

Rigorous peer-reviews are the basis of high-quality academic publishing. Due to the great efforts of our reviewers, *EJIHPE* was able to maintain its standards for the high quality of its published papers. Thanks to the contribution of our reviewers, in 2021, the median time to first decision was 18 days and the median time to publication was 44 days. The editors would like to extend their gratitude and recognition to the following reviewers for their precious time and dedication, regardless of whether the papers they reviewed were finally published:
Abreu, WilsonLópez-Cordero, RafaelAckova, DarinkaLópez-Gil, José FranciscoAlbendín-García, LuisLorenzo Lledó, AlejandroAlbornoz-Cabello, ManuelMacken, MarianAnastasiou, SophiaMalinauskas, RomualdasAparisi, DavidManeiro, LorenaArafat, YasirMaraffi, SabinaAvgerinos, EvgeniosMarín-Marín, José AntonioBacchini, DarioMartínez Ramón, Juan PedroBai, LiMartínez, Juan PedroBalgiu, Beatrice AdrianaMartínez-Sánchez, ÁngelBao, DatMaruta, MichioBarana, AliceMatud, PilarBeecroft, RichardMediavilla, MauroBishop, JonathanMirazchiyski, PlamenBolondi, GiorgioMonacis, LuciaBozkurt, ArasMoral-García, JoséBrkic, Faris F.Morenas Martín, JesúsCaldeiro Pedreira, Mari CarmenMorgan, HaniCaneiras, CátiaMoutsios-Rentzos, AndreasCarcedo De Andrés, Bruno P.Mucci, NicolaCastellary López, MacarenaMüller, PatrickCeccato, IreneMustafa, GhulamČeněk, JiříMytych, JenniferCerbara, LoredanaNajjar, MohammadChen, Chin-LingNavarro, RaulChristiansen, AndrésNikolopoulou, KleopatraCinganotto, LetiziaNobari, HadiColomer Feliu, JordiNúñez Pérez, José CarlosColomer, Juan CarlosOlivieri, GiulioCommodari, ElenaOmar, Syed HarisCrisp, DimityOprea, BogdanCrookes, GrahamOpydo-Szymaczek, JustynaDi Giacomo, DinaParra-Meroño, María ConcepciónDias, GonçaloPaszkowski, ZbigniewDíaz Noguera, María DoloresPatrinos, Harry A.Döring, NicolaPerak, BenediktDruica, ElenaPereira, HenriqueDueñas, Jorge-ManuelPetcu, Monica AurelianaĎuriš, ViliamPiñeiro, IsabelDuursma, Elisabeth E.Piotrowski, AndrzejElbattah, MahmoudPlasman, JayElhady, MarwaPlatania, SilviaEstévez, Jesús F.Popczyk, WojciechEstevez-Casellas, CordeliaQuaicoe, James SunneyFekete Farkas, MariaRachiotis, GeorgeFérnandez-Batanero, Jose M.Ramadas, AmuthaFernández-Prados, Juan SebastiánRathi, NehaFernández-Sogorb, AitanaRhee, Seung-YoonFrancesco, PuppoRivera Camino, JaimeFu, Albert QianyiRobitzsch, AlexanderFuster, Mª Carmen SánchezRodríguez, FranciscoGagatsis, AthanasiosRomano, LucianoGaman, Mihnea-AlexandruRosen, Mitchel A.Garcia, CarmeRuiz-Nuñez, CarlosGarcía-Perales, RamónSabelnikova, NataliaGiacomo, Dina DiSalavera, CarlosGimenez, GregorioSánchez Ruiz, Luis ManuelGrosso, GiuseppeSantiago Campión, RaúlHayward, Richard DavidSchumm, WalterHeil, JeanneSchwarz, EliHeinz, ManuelaShin, GisooHellemans, KimSiegmund, AlexanderHooshyar, DanialSingh, JitendraHua, JieStefani, LauraHunt, JodiStein, Sarah J.Ilieva, GalinaStephenson, Max O.Ivan, LoredanaStoeffler, KristinJia, YuaneStreit, JessicaKamke, KristynStuss, Magdalena M.Kaplan, SethSuárez, Mónica LuqueKelly, KimberlySungur, EnginKhubchandani, JagdishSunindijo, RizaKlímová, BlankaTanhan, AhmetKoch, MarthaTárraga Mínguez, RaúlKono, MasanoriTestoni, InesKonstantinov, Vsevolod V.Tomasik, Martin J.Kreuzfeld, SteffiTrimmer, KarenKrok, DariuszTsagkaris, ChristosKrpec, RadekValverde-Berrocoso, JesúsKrysinka, KarolinaVanduhe, Vanye ZiraKuliga, SaskiaVasconcelos, CarlosKummitha, RamaVîrgă, DeliaLabrosciano, ClementineVišnjić, AleksandarLavega-Burgués, PereWinston, Bruce E.Ledda, CaterinaWolfgang Kallus, K.Lee, BeatriceWu, MiaozongLeón, Samuel P.Wylie, RuthLeyton Roman, MartaXu, GuifengLiutsko, LiudmilaYang, XiaoLopez-Bueno, RubenŽižek, Simona Šarotar


